# Neutrophil Extracellular Trap Markers in Post Mortem Lung Biopsies from COVID-19 Patients

**DOI:** 10.3390/ijms26168059

**Published:** 2025-08-20

**Authors:** Mariana Collete, Thiago Rodrigues dos Santos, Natan de Araújo, Ana Paula Camargo Martins, Seigo Nagashima, Caroline Busatta Vaz de Paula, Cleber Machado-Souza, Lucia de Noronha

**Affiliations:** 1 Postgraduate Program of Health Sciences, School of Medicine and Life Sciences, Pontifical Catholic University of Paraná—PUCPR, Rua Imaculada Conceição, 1155-Prado Velho, Curitiba 80215-901, PR, Brazil; collete.mariana@pucpr.br (M.C.); natandearaujo@outlook.com.br (N.d.A.); 2 Postgraduate Program of Biotechnology Applied to Child and Adolescent, Faculdades Pequeno Príncipe, Health Instituto de Pesquisa Pelé Pequeno Príncipe, Av. Silva Jardim, 1632, Curitiba 80250-200, PR, Brazil; thiagorsantos.ippp@gmail.com (T.R.d.S.);; 3 Laboratory of Experimental Pathology, School of Medicine and Life Sciences, Pontifical Catholic University of Paraná—PUCPR, Rua Imaculada Conceição, 1155-Prado Velho, Curitiba 80215-901, PR, Brazil

**Keywords:** COVID-19, SARS-CoV-2, histopathology, lung, neutrophils, NETs

## Abstract

Severe Acute Respiratory Syndrome Coronavirus 2 (SARS-CoV-2), the causative agent of COVID-19, spread rapidly across the globe in 2020, with most countries experiencing two distinct waves of infection. In Brazil, the second wave was marked by the emergence of the P.1 (Gamma) variant, which disproportionately affected younger individuals and was associated with increased mortality. This study aimed to evaluate the epidemiological profile and post mortem histopathological lung findings, correlate them with laboratory results, and compare the first and second waves of COVID-19. To investigate neutrophil extracellular traps (NETs), we performed immunohistochemistry for citrullinated histone H3 (cit-H3) and myeloperoxidase (MPO). Our cohort included patients who died in the intensive care unit (ICU) of a single center in southern Brazil. The study included 42 patients, 24 from the first wave and 18 from the second, who died between March 2020 and August 2021. Laboratory data included complete blood counts and D-dimer levels. Histopathological analyses were conducted using H&E-stained slides and reviewed independently by two blinded pathologists. MPO and cit-H3 immunohistochemistry were performed to evaluate NETs markers. All cases exhibited varying degrees of inflammation and diffuse alveolar damage (DAD), with frequent microvascular thrombi. Neutrophilic infiltration was significantly higher in the second wave. Additionally, cases with intense neutrophilic infiltration showed a stronger association with thrombosis. NETs were identified in 10 cases. No significant correlation was found between histopathological findings, NETs, and laboratory blood count results. The histopathological findings were consistent with those reported globally. The second wave of COVID-19 showed higher neutrophilic infiltrate in the lung tissue. Neutrophils play a key role in the inflammatory response and NET formation might indicate an increased risk of mortality. Further studies can consider NET-targeted therapies as potential strategies.

## 1. Introduction

In Brazil, SARS-CoV-2 began spreading in early 2020. The first peak in mortality and new cases occurred between June and August of that year, followed by a decline in hospitalizations. However, the curve began to rise again in November 2020, leading to a second wave that peaked between April and May 2021, marked by a significant increase in infection rates and mortality [1,2].

Once in the lungs, SARS-CoV-2 triggers a complex network of molecular mechanisms that lead to systemic inflammation. This occurs through the activation of pneumocytes, leukocytes, macrophages and endothelial cells—a process known as a “cytokine storm” [3,4,5,6].

The predominant histopathological feature in fatal COVID-19 cases is diffuse alveolar damage (DAD), often accompanied by alveolar edema, hyaline membrane formation, varying degrees of inflammatory infiltrates, type II pneumocyte hyperplasia, and fibrosis. In addition to findings consistent with acute respiratory distress syndrome (ARDS), several studies have also reported other significant lesions, particularly microvascular injury characterized by capillaritis, microthrombi, and superimposed bronchopneumonia, often associated with bacterial infection [7,8,9,10,11].

Neutrophilia and elevated serum levels of neutrophil chemoattractants have been observed in severe cases of COVID-19 [12]. An elevated neutrophil-to-lymphocyte ratio (NLR) has been identified as an independent predictor of severe COVID-19 [13].

In response to inflammatory mediators—whether originating from the lungs or from distant organ injury—circulating neutrophils (polymorphonuclear leucocytes) become activated and are retained in the pulmonary capillary bed. They subsequently migrate across the endothelium, through the interstitium and epithelium, and into the alveolar space, contributing to local tissue dysfunction and destruction through the release of histotoxic mediators such as neutrophil extracellular traps (NETs) [14,15].

NETs are web-like structures composed of fragmented DNA bound to histones, along with granule enzymes such as neutrophil elastase (NE), myeloperoxidase (MPO), and cathepsin G. Initially described as microbicidal mechanisms, NETs are capable of ensnaring and killing pathogens [16].

NETosis refers to the formation of NETs associated with neutrophil cell death [17]. To date, two distinct mechanisms of NET formation have been identified: the primary pathway, known as suicidal NETosis, is a slow, lytic process that results in neutrophil death; the second, termed vital NETosis, allows neutrophils to release NETs while preserving their viability and continuing functions such as crawling and phagocytosis [18].

The function of NETs can be considered a ‘double-edged sword’ [19]. While neutrophil migration into the lung without activation does not cause tissue injury, uncontrolled NET formation contributes to the pathogenesis of various diseases [15,20,21].

Emerging evidence suggests that NET formation may be a key driver of severe COVID-19 cases [21,22,23]. It has been demonstrated that neutrophils activated by SARS-CoV-2 can release NETs, contributing to lung epithelial cell damage [24]. In this context, uncontrolled NETosis triggered by SARS-CoV-2 infection may promote thrombosis and exacerbate the ‘cytokine storm.’ Additionally, the high concentration of proteases released during this process is toxic to endothelial and epithelial cells, leading to severe multi-organ tissue damage [22].

Our study aims to help to elucidate the mechanisms of SARS-CoV-2 infection in the lung by analyzing histopathological findings from post mortem lung biopsies of COVID-19 patients, correlating these with laboratory results, and comparing cases from the first and second waves of the pandemic. We also investigate the presence of NETs using immunohistochemistry.

## 2. Results

### 2.1. Epidemiology and Demographical Information

A total of 42 cases were included in the study, comprising 30 males (71%) and 12 females (29%). The mean age was 65.6 years (sd ± 14.6), with a range from 30 to 96 years. The duration of hospitalization—from admission to death—ranged from 1 to 39 days, with a mean stay of 16.6 days (sd ± 8.83) (Supplementary Table S1). Of these, 24 patients died during the first wave of COVID-19, and 18 patients died during the second wave (Table 1).

### 2.2. Serum Tests: Platelets, Leucocytes, Neutrophils, D-Dimer and Neutrophil-Lymphocyte Ratio

Complete blood counts were obtained on the first day of hospitalization (Day 1) for all patients (*n* = 42), and within the final 24 h of hospitalization or prior to death (Day F) for 41 patients. The number of patients with neutrophilia increased from 19 on Day 1 to 34 on Day F. On Day F, none of the patients exhibited leukopenia; 13 had thrombocytopenia, and 30 presented with leukocytosis (Table 2). The mean neutrophil count increased from 8.776/μL to 18.012/μL, while the mean leukocyte count rose from 10.056/μL to 20.087/μL (Supplementary Table S2).

D-dimer levels exceeded 500 ng/mL in 26 of 30 patients (with available data) on Day 1, and in all 8 patients with available data on Day F (Table 2). Notably, one patient’s D-dimer level rose from 425 ng/mL on Day 1 to 7.384 ng/mL after 12 days of hospitalization. Among the 26 patients with elevated D-dimer levels, 12 also exhibited neutrophilia (Supplementary Table S1).

The neutrophil–lymphocyte ratio (NLR) ranged from 4.3 to 98.5. The median values in the first day of hospitalization was 15.8 with a standard deviation of 12.7. On the final 24 h before death, the median value was 23.9 with a standard deviation of 22.3.

### 2.3. Histopathology Findings

Histopathological examination of lung sections, conducted independently by two blinded pathologists, revealed inflammatory reactions in all cases (100%), ranging from mild to intense and affecting all compartments of the lung:interstitial, vascular, alveolar, and bronchiolar. Mononuclear leukocytes were present in all cases, while polymorphonuclear leukocytes were observed in the interstitial compartment in most cases (*n* = 37). Acute bronchopneumonia associated with bacterial colonies was identified in one case.

Diffuse alveolar damage (DAD) was identified in 41 cases (97%), presenting in various stages: 14 cases (33.3%) in the exudative phase, 14 (33.3%) in the proliferative phase, and 13 (30.9%) in the fibrotic phase. As typically observed in DAD, hyaline membranes were evident in 32 cases (76%). Fibrosis, at least focally, was present in 33 cases (78%), and pneumocyte hyperplasia was observed in 39 cases (92.8%).

**Figure 1:**
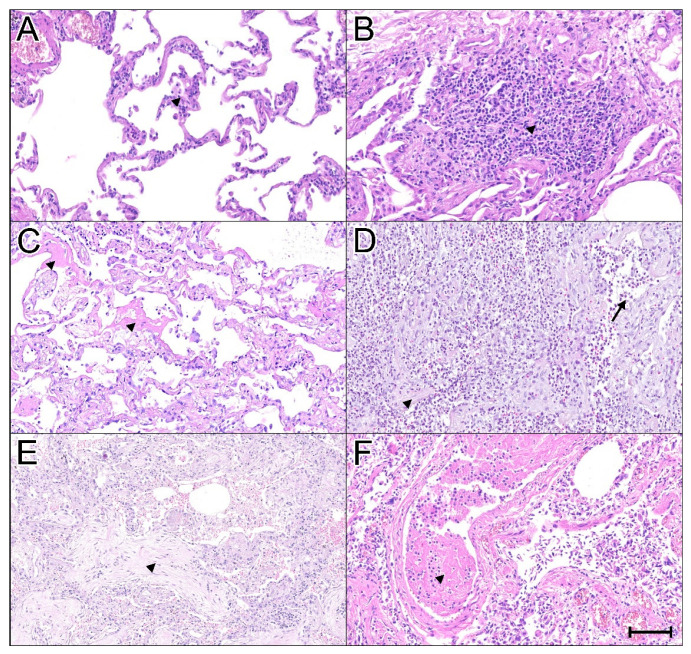
Summary of key histopathological findings in lung tissue samples from 42 COVID-19 patients. A variety of inflammatory patterns and different phases of diffuse alveolar damage (DAD) were identified in lung tissue from COVID-19 patients (Hematoxylin–Eosin, 100× magnification). (**A**) Mild lymphocytic septal infiltrate (indicated by arrowhead). (**B**) Intense septal lymphocytic infiltrate with scattered neutrophils (arrowhead). (**C**) Exudative-phase DAD with mild inflammation and hyaline membranes (arrowhead). (**D**) Proliferative-phase DAD with early fibroblastic proliferation (arrowhead) and intense neutrophilic infiltrate in septal and alveolar spaces (arrow). (**E**) Fibrotic-phase DAD showing moderate inflammation—predominantly lymphocytes—and fibrosis (arrowhead). (**F**) Capillaritis and thrombus with neutrophils in an arteriole (arrowhead). Scale bar = 100 μm.

In the vascular compartment, capillaritis was observed in 22 cases (52.3%), and microvascular thrombi were present in 28 cases (66.6%), including 9 cases with thrombi measuring 1 mm or more. Neutrophil-rich thrombi were identified in 14 cases (33.3%) (Supplementary Table S1). The main histopathological findings are illustrated in Figure 1.

### 2.4. Comparison of Histopathological Findings

Among patients with absent or mild interstitial infiltration of polymorphonuclear leukocytes, 15 (55.6%) presented with thrombosis, while 12 (44.4%) did not. In contrast, among those with moderate to intense infiltration, the majority (86.7%) had thrombosis, and only two patients (13.3%) did not (*p* = 0.04). These results are summarized in Table 3.

Patients with a greater degree of mononuclear infiltrate, particularly lymphocytes, exhibited more moderate to intense fibrosis (*p* < 0.01; Table 4).

No statistically significant differences were observed for the following comparisons: phase of DAD versus thrombosis (*p* = 0.379), fibrosis versus thrombosis (*p* = 0.346), and mononuclear inflammation versus thrombosis (*p* = 0.213).

### 2.5. Histopathological Findings and Duration of Hospitalization from Admission to Death

In our cohort, 21 patients (50%) were hospitalized for more than 14 days, while the remaining 21 (50%) had hospital stays of 14 days or less (Table 5). Patients with longer hospitalizations were more likely to present with the fibrotic-phase DAD (*p* = 0.047), as well as a higher prevalence of mononuclear (lymphocytic) inflammation.

### 2.6. Comparative Analysis of the First and Second COVID-19 Waves

Group 1 *(n* = 24) consisted of patients from the first wave of COVID-19, while Group 2 (*n* = 18) included patients from the second wave. The mean age of patients in Group 2 was significantly lower than that of Group 1 (57.2 years vs. 71.9 years, respectively). Hospitalization duration showed only minor differences between the groups. Notably, neutrophilic infiltration was significantly greater in the second-wave cases (*p* = 0.004). There was no statistically significant difference in serological results between the two groups. Clinical and pathological findings for both groups are summarized in Table 6.

D-dimer values were available for 30 patients on Day 1 of hospitalization and for 8 patients on Day F. Results from Day 1 are summarized in Table 7. Prior to death, all 8 patients (100%) had elevated D-dimer levels (above 500 ng/mL).

### 2.7. Immunohistochemistry of NETs Markers

Most cases demonstrated abundant positivity for citrullinated histone H3 (cit-H3) in various cell types, including leukocytes, epithelial cells, and endothelial cells, indicating chromatin decondensation. Myeloperoxidase (MPO)-positive cells were also present, though to a lesser extent. Intravascular MPO-positive neutrophils were observed in 40 of the 42 cases.

**Figure 2:**
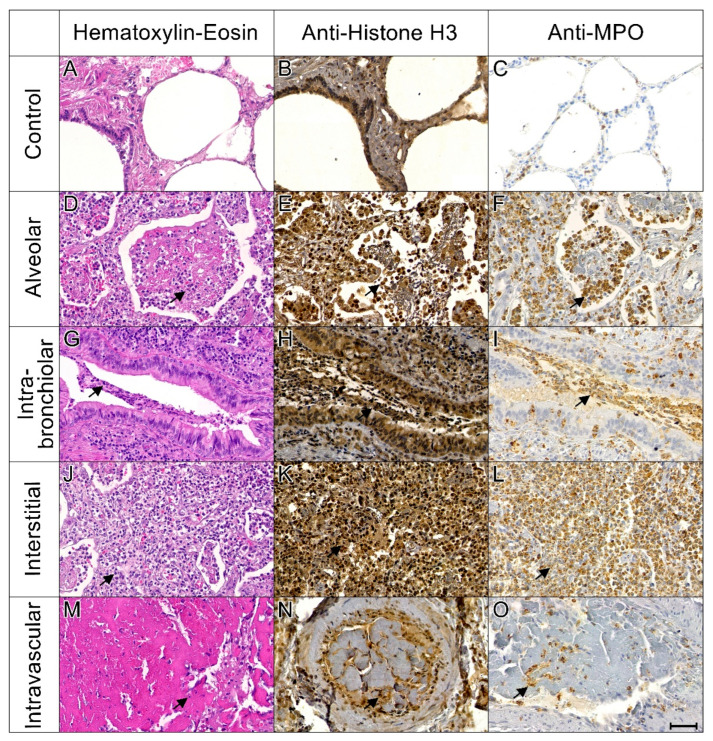
Histopathological and immunohistochemical evaluation of NETs localization. Columns represent H&E staining, immunostaining for citrullinated histone H3 (Anti-cit-H3), and myeloperoxidase (Anti-MPO), from left to right. The first row (images (**A**–**C**)) shows lung tissue from a control case, with no detectable cit-H3 or MPO staining in neutrophils. The following rows illustrate distinct patterns of NET localization: alveolar (images **D**–**F**), intra-bronchiolar (**G**–**I**), interstitial (**J**–**L**), and intravascular (**M**–**O**). In all NET-positive areas, H&E sections show increased inflammatory cell infiltration, with corresponding cit-H3 and MPO positivity (indicated by arrows), consistent with NET formation. Images in 200× magnification. Scale bar: 50 μm.

Neutrophils co-expressing MPO and cit-H3, particularly in areas with neutrophil aggregates and extracellular traps identifiable on Hematoxylin–Eosin (H&E) staining, were consistent with the presence of NETs. These NET structures were observed in 10 COVID-19 cases, involving all lung compartments (Figure 2). In contrast, no NETs were detected in the control group (*n* = 8); only focal MPO and cit-H3 positivity was observed.

### 2.8. Clinical Information, Serum Results and Histopathology Findings of Patients with Evidence of NETs

Among the patients with evidence of NETs (*n* = 10), ages ranged from 30 to 75 years. The duration of hospitalization varied between 8 and 39 days. As expected, based on histopathological analysis, Group 2 showed a higher frequency of NETs (7 out of 18; 38.8%) compared to Group 1 (3 out of 24; 12.5%). DAD was observed in various phases, most of which were highly inflamed, with prominent intra-alveolar neutrophilic and lymphocytic infiltrates. Microthrombi were identified in eight cases, including three with thrombi measuring more than 1 mm. Clinical data and histopathological features are summarized in Table 8 and Supplementary Table S1.

Blood count results were available for all patients with evidence of NETs (*n* = 10) on Day 1 of hospitalization, and for nine of them on the final day before death (Day F). Thrombocytopenia was observed in five patients at admission and in two patients at the final assessment. Leukocytosis was present in three patients on Day 1 and increased to nine patients by the Day F. Similarly, neutrophilia was noted in four patients at admission and in nine patients at the end of hospitalization. The mean neutrophil count increased from 8.876/μL to 20.294/μL, while the mean leukocyte count rose from 10.470/μL to 23.200/μL. However, there was no statistically significant association between the presence of NETs and the range of neutrophil counts or D-dimer levels. D-dimer levels were above 500 ng/mL in five of the seven patients with available data, ranging from 290 ng/mL to 1.652 ng/mL (Table 9).

## 3. Discussion

### 3.1. Histolopathologic Findings, Trombosis and the Role of Neutrophils

We analyzed 42 post mortem specimens from patients who died in the ICU. Our findings align with previous reports in the literature and are predominantly characterized by diffuse alveolar damage (DAD) in various stages (*n* = 41), varying degrees of inflammation (*n* = 42), and the presence of microthrombi (*n* = 28) [5,9,10,11,25].

Multiple genetic pathways involved in pulmonary thrombosis have been shown to be activated in the early stages of critical COVID-19 illness [26,27]. In a review combining data from several cases, 72% (163/227) had microthrombi [28]. In our cohort, microthrombi were present in 67% of cases, though this may be underestimated due to the limited size of the tissue samples analyzed.

Our group previously described the presence of activated endothelial cells and elevated levels of biomarkers associated with vascular injury, including ICAM-1, ANGPT-2, and IL-1β, as well as increased expression of angiogenesis markers such as VEGF in fatal COVID-19 cases, compared to both H1N1 and control groups [29]. Supporting these findings, another study reported that the prevalence of alveolar capillary microthrombi in patients with COVID-19 was nine times higher than in those with influenza (*p* < 0.001) [30].

There remains some controversy over whether neutrophil infiltration in the lungs represents a primary innate immune response to COVID-19 or is the result of superimposed bacterial or fungal infections [9,27,28,31]. Widespread neutrophilic infiltration has been associated with severe COVID-19 in a series of 21 autopsies [25]. In our study, we observed varying degrees of neutrophilic infiltrates in the lungs, along with serum neutrophilia preceding death in most cases (*n* = 34). Notably, cases with moderate to severe neutrophilic infiltration were more likely to exhibit thrombosis (*p* = 0.04).

Histological features consistent with acute bronchopneumonia caused by bacterial infection were identified in one case, supported by the presence of bacterial granules. In a review of 603 COVID-19 autopsies, superimposed infections were reported in 27% of cases [6]. However, a study of 68 autopsies described a subset of patients who experienced rapid progression to severe respiratory failure without evidence of superinfection and without mechanical ventilation. In these cases, neutrophils were associated with alveolar wall injury, necrosis, and microthrombi, reinforcing the role of COVID-19 itself in driving lung injury [31].

The exudative phase of DAD was frequently found with shorter durations of illness [32] whereas prolonged cases of COVID-19 have been associated with progressive lung fibrosis and chronic inflammation [11,28]. In our cohort, when comparing fatal cases with more than 14 days of hospitalization to those with 14 days or less, patients with longer hospital stays showed a higher frequency of fibrosing-phase DAD (52% vs. 9%), moderate to severe mononuclear cell infiltrates (81% vs. 62%), moderate to severe neutrophilic infiltrates (42% vs. 28%), and microthrombi (52% vs. 38%). However, the overall frequency of thrombosis was not significantly correlated with the duration of hospitalization in this study.

### 3.2. Comparison Between the Two Waves of COVID-19

The second wave of the COVID-19 pandemic coincided with the global emergence and spread of viral variants exhibiting higher transmissibility than the original strain [33,34]. By December 2020, several variants of concern had been identified: the Alpha variant (B.1.1.7 and Q lineage) in the United Kingdom, the Beta variant (B.1.351) in South Africa, and the Gamma variant (P.1) in Brazil. By June 2021, the Delta variant (B.1.617.2 and AY lineage) had emerged in India, and the Epsilon variant (B.1.427 and B.1.429) was identified in the United States, followed by the Omicron variant (B.1.1.529), identified in South Africa and Botswana [1,35]. During Brazil’s second wave, there was a marked increase in deaths, particularly among individuals under 60 years old [36]. During the first and second waves of COVID-19 in Brazil, the highest hospital and ICU admission rates per 100,000 inhabitants were mainly concentrated in the states of Distrito Federal (DF), São Paulo (SP), Paraná (PR), and Rio Grande do Sul (RS) [37]. In Paraná, where this study was conducted, the Gamma variant became predominant in February 2021 [38], accompanied by increased fatality rates among young and middle-aged adults. In Rio Grande do Sul, one hospital reported that most cases (25 out of 27) were of the P.1 lineage of SARS-CoV-2 [39].

In Spain and most European countries, the second wave predominantly affected younger patients, who had shorter hospital stays and lower mortality rates [2,40]. In a global analysis involving 53 countries, 43 of them reported a lower mortality rate during the second wave, including Brazil [40]. However, that study defined the second wave as occurring after June 2020, a timeline that does not accurately represent the progression of the pandemic in Brazil, where the second wave began later.

In Italy, patients who died in the second wave had fewer comorbidities and superinfection [41]. An analysis of 35 complete autopsies of COVID-19 cases found that cerebral and systemic hemorrhages were more frequent during the first wave compared to the second wave [42].

In our study, patients from the second wave (Group 2) had a lower mean age compared to those from the first wave (Group 1) (52 years vs. 71 years), while the average duration of hospitalization did not differ significantly between groups (17 days vs. 15 days). Notably, Group 2 patients exhibited significantly higher neutrophilic infiltration (*p* = 0.004), reinforcing the role of neutrophils in COVID-19 pathogenesis and suggesting a possible link between intense neutrophilic inflammation and SARS-CoV-2 variants.

Few studies have investigated the histopathological response of neutrophils to the different SARS-CoV-2 variants of concern (VOCs). In vitro, the Delta spike protein induced significantly higher neutrophil activation compared to the Omicron spike and the ancestral strain. A higher concentration of circulating NETs was reported in the Delta variant, although blood neutrophil levels were similar between the Delta and Omicron groups [43].

In Syrian hamsters, a large number of macrophages and neutrophils were observed in the lungs of Gamma-infected animals. Most variants (Beta, Epsilon, Gamma, Delta, Omicron) exhibited similar histopathological findings, although Omicron-infected animals showed lower histopathology scores. The Beta and Gamma groups had higher viral titers compared to other variants [44]. In autopsies related to the COVID-19 B.1.617.2 Delta variant, the histopathological lung findings were consistent with those previously described in the COVID-19 literature [45].

To our knowledge, no prior studies have compared histopathological findings between the first and second COVID-19 waves in Brazil. Given the significant difference in neutrophilic infiltration, we hypothesize that the Gamma variant may trigger a stronger innate immune response.

### 3.3. NETs, Immunothrombosis and D-Dimer

Shi et al. demonstrated that calprotectin levels—a marker of neutrophil activation—were significantly higher in hospitalized COVID-19 patients who progressed to severe disease requiring mechanical ventilation (8039 ± 7031 ng/mL, *n* = 32) compared to those who remained free of intubation [46]. Additionally, levels of calprotectin and cell-free DNA positively correlated with peak platelet counts and were predictive of reduced oxygen efficiency [47].

It is well established that a SARS-CoV-2 infection can trigger an exaggerated immune response, with neutrophils playing a central role in tissue injury [23,48]. Activated neutrophils release various pro-inflammatory mediators—including interleukin-6 (IL-6), interleukin-8 (IL-8), interferon-γ (IFN-γ), and tumor necrosis factor-α (TNF-α)—as well as NETs [48]. While no other coronaviruses have previously been shown to induce NET formation [22], there is growing evidence that SARS-CoV-2 can stimulate neutrophils to release NETs via mechanisms dependent on ACE2 and serine protease activity [24]. NET formation may thus be driven by both a cytokine storm and, potentially, direct infection of neutrophils by the virus [24,49].

Moreover, SARS-CoV-2 may evade components of the innate immune system, potentially leading to chronic NET auto-stimulation, with effects resembling autoimmune disease [22]. Elevated circulating markers of NETs have been associated with poor clinical outcomes, defined by ICU admission and/or short-term mortality in COVID-19 patients [49], as well as with poor prognosis in influenza A infection [50]. Several additional studies have reported increased NET levels in both circulation and lung tissue, correlating with disease severity in COVID-19 [47,51,52,53].

Krinsky et al. studied 16 COVID-19 patients and found that paired samples taken during acute illness and convalescence revealed significantly lower NETosis induction after recovery [53]. Similarly, patients who showed clinical improvement had lower serum levels of citrullinated histone H3 (cit-H3) compared to those without improvement [54]. Furthermore, extracellular cit-H3 was exclusively detected in COVID-19 patients, but not in healthy controls [55]. These findings, derived from plasma analyses, strongly suggest that circulating NETs contribute to the systemic inflammatory response observed in COVID-19 and are not merely a consequence of mechanical ventilation-induced pulmonary stress [52].

NETs can be identified in formalin-fixed, paraffin-embedded (FFPE) tissue samples using immunohistochemistry (IHC) staining for citrullinated histone H3 (cit-H3) and myeloperoxidase (MPO), which allows for the unambiguous visualization of neutrophil granule-derived proteins and histones co-localizing with extracellular web-like structures [20,50]. In our study, we observed widespread histone and MPO positivity using this method. Co-localized staining for cit-H3 and MPO, with characteristic web-like morphology, was evident in lung tissues from 10 patients, consistent with the presence of NETs. These structures were observed in both intravascular and extravascular compartments, in agreement with previous studies that have identified NETs throughout various lung regions in fatal COVID-19 cases [24,49,56].

NETs were primarily associated with inflammatory interstitial lesions and airway involvement, while NET-prone primed neutrophils were found within the pulmonary microcirculation [56]. These findings further support the role of NETs in the pathogenesis of severe COVID-19.

Excessive neutrophil activation can lead to oxidative stress, local and systemic inflammation, and subsequent endothelial damage in capillaries, contributing to an increased risk of thrombotic events [10,48]. The presence of NETs within pulmonary thrombi, as reported in several studies, supports their role in immunothrombosis and coagulopathy in COVID-19 [10,11,19,49,57,58]. Although we did not observe a statistically significant correlation between NETs and immunothrombosis in our study, higher neutrophil infiltration was associated with a greater likelihood of thrombosis (*p* = 0.04).

Once MPO and cit-H3 were diffusely expressed in the epithelial and inflammatory cells in the lung, the evidence of NETs in 10 cases may be underestimated due to the small fragment analyzed. Nevertheless, the second wave of COVID-19, a period of the variant Gamma prevalence, had cases with higher neutrophilic infiltrate (*p* = 0.04) and more cases with evidence of NETs (70%).

Experimental models in mice have shown that histones can bind to the endothelial surface, promoting platelet aggregation and platelet-dependent thrombin generation. Concurrently, extracellular chromatin fibers act as scaffolds within the circulation, facilitating thrombus formation and disrupting blood flow. These findings suggest that NETosis can directly damage the endothelium and may sharply increase the risk of deep vein thrombosis [59].

Elevated D-dimer levels are frequently observed in severe COVID-19 [9,10,12,60,61] and have been positively correlated with NETosis induction [47,49,53]. A threshold of >1000 ng/mL has been associated with poor clinical outcomes [60,61]. Despite these associations, several studies—including ours—did not find a direct correlation between D-dimer levels and the presence of NETs. This discrepancy may reflect a lack of thrombus degradation in these patients [51]. However, it is important to note that none of the patients in whom NETs were identified in our cohort had D-dimer measurements taken within 24 h of death. Additionally, an evaluation of a hypercoagulable state showed that high levels of hypercoagulable rotational tromboelastometry were associated with clinical deterioration and increased risk of persistent symptoms in COVID-19 survivors [62].

### 3.4. Markers of Mortality in COVID-19

Several mortality markers have been studied in COVID-19. An increase in neutrophil count follows the inflammatory response, alongside the release of pro-inflammatory cytokines such as IL-6 and TNF-α by lymphocytes and endothelial cells. The progression of COVID-19 has been associated with elevated plasma levels of acute-phase reactants, including C-reactive protein (CRP), IL-6, D-dimer, and procalcitonin [3,6,63]. In a previous study, our group investigated cytokines such as IFN-γ, TNF-α, IL-4, IL-6, and IL-10 in blood samples, concluding that IFN-γ is an independent risk factor associated with mortality [64].

The neutrophil–lymphocyte ratio (NLR) has been reported as an independent risk factor for in-hospital mortality in COVID-19 patients [13,65]. In an analysis of 697 COVID-19 patients, high values of NLR, D-dimer, procalcitonin, IL-6, and CRP were predictive of mortality [63], with a proposed NLR cut-off of 9.9 [63]. In the present study, 74.3% (29 out of 39) of the patients had an NLR higher than 9.9 when measured 24 h before death. On the first day of hospital admission, a higher probability of death was observed in patients with an NLR above 4.82 [66]. In our cohort, 85.3% (35 out of 41) of patients had an NLR above 4.8 on Day 1.

Additionally, several markers have been repeatedly linked to COVID-19 severity and mortality. An analysis of four NET markers in serum found an association only between cfDNA and severity [67]. Considering these findings, although our cohort includes a small number of patients, we believe that NETs could serve as a potential marker of mortality, though they are not exclusive.

### 3.5. Final Considerations

In summary, we found higher neutrophilic infiltrate in the second wave of COVID-19 compared to the first wave, a period of predominance of the Gamma variant. There is evidence of NETs in 10 cases (23.8%). NETs were absent in the control group (*n* = 8), reinforcing the conclusion that NET formation is not merely a post mortem artifact [50].

Within the complex inflammatory environment, neutrophils play a significant role, but other cell types—such as macrophages, epithelial cells, and endothelial cells—are also critically involved [12,15,68,69,70].

This study has several limitations. First, the population was limited to patients who died with COVID-19 in the ICU of a single center, which may not be representative of the broader patient population. Moreover, the fact that pulmonary sample collection was performed through an invasive method (mini-thoracotomy) in an ICU setting limited the inclusion of a larger number of patients in the study. Second, despite independent reviews by two pathologists, histopathological interpretation remains partially subjective, and the semi-quantitative assessment of NETs is inherently limited by this subjectivity. Third, as this study relied on post mortem biopsies, only small lung tissue fragments were available for analysis, which may have led to sampling bias and missed pathological features present elsewhere in the lungs. Forth, due to financial constraints, D-dimer levels were not available for all patients. Finally, the failure to associate peripheral blood immune subsets with histopathological findings could be influenced by the kinetics of the inflammatory cells, influenced by the disease severity and drug administration [71].

Despite the limitations, our study provides robust histopathological evidence that contributes meaningfully to the understanding of COVID-19 pathology, particularly regarding the role of NETs and the innate immune response and supports the growing recognition of disease heterogeneity in in COVID-19.

## 4. Materials and Methods

### 4.1. Collection of Post Mortem Samples and Clinical Information

This study was approved by the National Research Ethics Committee (Comitê Na- cional de Ética em Pesquisa—CONEP), under the numbers 3.944.734/2020 (COVID-19 patients) and 2.550.445/2018 (CONTROL patients). The methodology was performed following relevant guidelines and regulations. The participants’ legal representatives allowed the use of tissue samples for this study.

COVID-19 group:

The clinical data of patients who died of COVID-19 (*n* = 42) came from the medical records of the intensive care unit (ICU) of the Marcelino Champagnat Hospital, in Curitiba, Brazil. Individuals were subcategorized into two groups according to the period of hospitalization and death: Group 1 consists of patients from the first wave (*n* = 24) who died between April and August 2020, and Group 2 consists of patients from the second wave (*n* = 18) who died between March and June 2021.

The post mortem lung samples, measuring 3 × 3 cm, were obtained through left anterior mini-thoracotomy with upper left lobe segment resection. The time from death to tissue collection was less than 4 h, as a result of the elevated turnover rate of ICU beds during the critical phase of the pandemic.

All COVID-19 patients were tested for SARS-CoV-2 using nasopharyngeal swabs, with positive results confirmed by RT-qPCR.

The lung samples were fixed in buffered formalin for 12–24 h, followed by dehydration, clearing, and paraffin embedding. The formalin-fixed paraffin-embedded (FFPE) tissue blocks were sectioned into 4 µm thick slices and stained using the Hematoxylin–Eosin (H&E) method (Harris Hematoxylin: NewProv, Cod. PA203, Pinhais, Brazil; Eosin: BIOTEC Reagentes Analíticos, Cod. 4371, Pinhais, Brazil).

Laboratory evaluations included D-dimer levels, and neutrophil, leukocyte, and platelet counts. Neutrophilia was defined as a neutrophil count greater than 8.000 cells/μL, leukocytosis as a leukocyte count exceeding 10.000 cells/μL, and thrombocytopenia as a platelet count below 150.000 cells/μL.

Patients who died during the first wave (March–August 2020) were classified as Group 1, whereas Group 2 consisted of patients who died during the second wave (February–June 2021).

CONTROL group:

The control group (*n* = 8; 1 female, 7 males; age range = 23–54) consisted of lung tissue samples collected during autopsies conducted at the Forensic Medicine Institute in Curitiba, Brazil. All individuals in this group died from traumatic causes and had not been hospitalized prior to death, minimizing potential confounding factors such as cytokine elevation or other molecular changes that could affect immunohistochemical findings. Post mortem lung samples were formalin-fixed and paraffin-embedded (FFPE) for Hematoxylin–Eosin (H&E) staining and histopathological analysis. All control patients tested negative for SARS-CoV-2 via RT-qPCR on nasopharyngeal swabs.

Based on FFPE lung samples from the COVID-19 and control groups, multi-sample paraffin tissue blocks (Tissue Microarray, TMA) were constructed to facilitate the execution of the histochemical and immunohistochemical assays. Three cylindrical fragments (chosen from affected areas guided by H&E staining), measuring 0.3 cm in diameter, were removed from the original FFPE blocks and reorganized into one new multi-sample paraffin blocks in order to reduce expenses, resource consumption, and analysis time.

### 4.2. Immunohistochemistry Assay

Immunohistochemical staining was performed in TMA samples to identify markers of NETs, using the following primary antibodies: anti-cit-H3 (code SAB4500352, polyclonal/rat/mouse, dilution 1:100, Sigma Aldrich, Burlington, MA, USA) and anti-MPO (code HPA061464, polyclonal/human, dilution 1:200, Sigma Aldrich, Burlington, MA, USA). Briefly, the technique was processed in an overnight incubation protocol for primary antibodies in a humid chamber between 2 and 8 °C. The secondary polymer (Mouse and Rabbit Specific HRP/DAB IHC Detection Kit—Micro-polymer, Abcam, ab236466, Cambridge, UK) was applied to the material tested for 20 min at room temperature. The technique was revealed by adding the 2, 3, diamino-benzidine complex + hydrogen peroxide substrate, the time required was indicated by it turning brown, and the counterstaining was performed with Harris hematoxylin. The reactivity of the positive control confirmed the results.

### 4.3. Histopathological Analysis

Two blinded pathologists independently conducted the histopathological evaluations without access to clinical data. TMA slides were examined using a BX50 optical microscope (OLYMPUS, Tokyo, Japan). Cases with discordant interpretations were reviewed jointly and classified by consensus.

Histological parameters were evaluated across three compartments, with characteristics recorded based on the most affected microscopic fields: The Interstitial Compartment was assessed for mononuclear inflammatory infiltrate, neutrophilic inflammatory infiltrate, and fibrosis, each categorized as mild, moderate, or intense. The Alveolar Compartment was evaluated for mononuclear inflammatory infiltrate, neutrophilic inflammatory infiltrate, and edema, also categorized as mild, moderate, or intense. Presence or absence of hyaline membranes and pneumocyte hyperplasia was recorded. The Vascular Compartment was examined for thrombosis, distinguishing between microthrombi (<1.0 mm) and large thrombi (≥1.0 mm), as well as for neutrophil-rich thrombi.

Diffuse alveolar damage (DAD) was classified into three stages: The Exudative Phase was characterized by intra-alveolar edema, hyaline membrane formation, type II pneumocyte hyperplasia, and varying degrees of hemorrhage, fibrin deposits, and inflammatory infiltrates. The Proliferative Phase was defined by the presence of fibroblast proliferation or mild fibrosis in addition to features observed in the exudative phase. The Fibrotic Phase was marked by moderate to severe fibrosis, septal thickening, and minimal or absent hyaline membranes. When multiple patterns were present in a sample, the most advanced stage observed was recorded.

Bronchiolar inflammation was registered, whether present or absent.

### 4.4. Statistical Analysis

Histopathological and clinical data were recorded in an Excel spreadsheet and analyzed using the software JMP (TM) Pro 14.0.0. (SAS Institute, Cary, NC, USA). The normality condition was evaluated using the Shapiro–Wilk test. Descriptive analyses were used to determine the patients’ epidemiological and clinical features. Continuous variables were presented using mean ± standard deviation and categorical variables were expressed as absolute valor and percentages. Comparisons were performed using Chi-square or Fisher’s exact test for categorical variables. A *p*-value < 0.05 was considered statistically significant.

## 5. Conclusions

The histopathological features observed in 42 post mortem lung biopsies were consistent with those reported globally, emphasizing the significant role of neutrophils in the pathophysiology of fatal COVID-19 and their contributions to tissue damage, inflammation, and thrombosis.

By comparing cases from the first and second waves of the pandemic, we found the more intense neutrophilic infiltrate in the second phase. We raise the hypothesis that SARS-CoV-2 variants (especially Gamma variant) may induce a more pronounced neutrophilic response than the original strain.

In our cohort, immunohistochemical staining for cit-H3 and MPO, along with histological analysis, confirmed the presence of NETs in 10 cases (23%), supporting that neutrophil could influence the adaptive immune response through the formation of NETs [29]. These findings suggest that NET formation may be related to mortality and NET-targeted therapies could be considered as potential strategies to mitigate the excessive tissue damage and thrombosis observed in severe COVID-19 [21,50].

Overall, this study offers valuable insights into lung pathology in fatal COVID-19 cases and underscores the need for continued research into host immune responses—particularly neutrophil-driven inflammation—and the effects of viral evolution on immunopathology. A deeper understanding of these immunologic mechanisms and the disease’s heterogeneity is essential for improving clinical management and guiding more effective therapeutic interventions.

## Figures and Tables

**Table 1:** Epidemiology and clinical information.

Clinical Information	Value
Total number of cases	42
Gender—male	30 (71%)
Gender—female	12 (29%)
Age (years)	65.6 ± 14.6 ^a^
From admission to death (days)	16.6 ± 8.83 ^a^
Death during the first wave	24 (57.1%)
Death during the second wave	18 (42.9%)

Categorical variables expressed as absolute number (frequency). Continuous variables expressed by mean ± standard deviation (sd) ^a^.

**Table 2:** Hematological parameters at admission (Day 1) and in the final 24 h of hospitalization (Day F).

Variable	Day 1 (*n* = 42)	Day F (*n* = 41)
Neutrophilia (patients)	19/42 (45.2%)	34/41 (82.9%)
Leukocytosis (patients)	15/42 (35.7%)	30/41 (73.1%)
Thrombocytopenia (patients)	14/42 (33.3%)	12/41 (29.3%)
D-dimer > 500 ng/mL (patients)	26/30 (86.7%)	8/8 (100%)
D-dimer > 1000 ng/mL (patients)	15/30 (50%)	7/8 (87.5%)

Categorical variables expressed as absolute number/number of patients (frequency).

**Table 3:** Comparison between PMN interstitial infiltrate and thrombosis.

PMN Infiltrate	Thrombosis Present	Thrombosis Absent	*p*-Value
Absent/Mild (*n* = 27)	15 (55.6%)	12 (44.4%)	0.04
Moderate/Intense (*n* = 15)	13 (86.7%)	2 (13.3%)	

PMN = polymorphonuclear leucocytes. Categorical variables expressed as absolute number (frequency). Fisher’s exact test *p*-values  <  0.05 considered statistically significant.

**Table 4:** Comparison between MN interstitial infiltrate and fibrosis.

MN Infiltration	Fibrosis Absent/Mild	Fibrosis Moderate/Intense	*p*-Value
Absent/Mild	13 (81.2%)	3 (18.8%)	<0.01
Moderate/Intense	5 (19.2%)	21 (80.8%)	

MN = mononuclear leucocytes. Categorical variables expressed as absolute number (frequency). Fisher’s exact test *p*-values <  0.05 considered statistically significant.

**Table 5:** Histopathological findings stratified by hospitalization duration from admission to death.

Histopathological Findings		Duration of Hospitalization—From Admission to Death	
		14 Days or Less (*n* = 21)	More than 14 Days (*n* = 21)
Inflammatory infiltrate in interstitial compartment	MN—Absent/Mild	12 (57%)	4 (19%)
	MN—Moderate/Intense	9 (43%)	17 (81%)
	PMN—Absent/Mild	14 (66.7%)	13 (62%)
	PMN—Moderate/Intense	7 (33.3%)	8 (38%)
DAD—phase	DAD—absent	1 (4.8%)	0
	DAD—exudative phase	10 (47.6%)	4 (19%)
	DAD—proliferative phase	8 (38%)	6 (28.6%)
	DAD—fibrotic phase	2 (9.6%)	11 (52.4%)
Vascular compartment	Thrombi	8 (38%)	11 (52.4%)
	Thrombi (≥1 mm)	4 (19%)	5 (23.8%)
	absence of thrombi	9 (43%)	5 (23.8%)

MN = mononuclear leucocytes. PMN = polymorphonuclear leucocytes. DAD = diffuse alveolar damage. Categorical variables expressed as absolute number (frequency).

**Table 6:** Clinical, serological, and histopathological findings in the first and second COVID-19 waves.

	Group 1 (First Wave) n = 24	Group 2 (Second Wave) n = 18	*p*-Value
Gender (male) ^a^	15 (62.5%)	15 (83.3%)	
Gender (female) ^a^	9 (37.5%)	3 (16.7%)	
Age (years) ^b^	71.9 ± 12.4	57.2 ± 13.3	
Duration of Hospitalization (days) ^b^	15.8 ± 10.2	17.6 ± 6.6	
Thrombocytopenia ^a^			
Present	8 (34.8%)	4 (22.2%)	
Absent	15 (65.2%)	14 (77.8%)	*p* > 0.333
Leukocytosis ^a^			
Present	16 (66.7%)	14 (77.8%)	*p* > 0.262
Absent	8 (33.3%)	4 (22.2%)	
Neutrophilia ^a^			
Present	19 (79.2%)	15 (83.3%)	*p* > 0.679
Absent	5 (20.8%)	3 (16.7%)	
NLR ^b^	22.0 ± 15.4	26.6 ± 29.9 (*n* = 16)	
MN interstitial infiltrate ^a^			
Mild	10 (41.6%)	6 (33.3%)	*p* = 0.174
Moderate	7 (29.2%)	5 (27.7%)	
Intense	7 (29.2%)	7 (39%)	
PMN interstitial infiltrate ^a^			
Absent	5 (20.9%)	1 (5.6%)	*p* = 0.004
Mild	15 (62.5%)	6 (33.3%)	
Moderate	4 (16.6%)	10 (55.5%)	
Intense	0	1 (5.6%)	
Interstitial fibrosis ^b^			
Present	18 (75%)	15 (83.3%)	*p* = 0.708
Absent	6 (25%)	3 (16.7%)	
MN alveolar infiltrate ^a^			
Absent	0	1 (5.6%)	*p* = 0.194
Mild	9 (37.5%)	4 (22.2%)	
Moderate	9 (37.5%)	9 (50%)	
Intense	6 (25%)	4 (22.2%)	
PMN alveolar infiltrate ^a^			
Absent	8 (33.3%)	1 (5.6%)	*p* = 0.0049
Mild	11 (45.8%)	6 (33.3%)	
Moderate	3 (12.5%)	3 (16.7%)	
Intense	2 (8.4%)	8 (44.4%)	
Hialine membrane ^b^			
Present	19 (79.1%)	13 (72.3%)	*p* = 0.601
Absent	5 (20.9%)	5 (27.7%)	
Diffuse alveolar damage ^a^			
Absent	0	1 (5.6%)	*p* = 0.047
DAD—exudative phase	9 (37.5%)	5 (27.7%)	
DAD—proliferative phase	11 (45.8%)	3 (16.7%)	
DAD—organization phase	4 (16.6%)	9 (50%)	
Vascular thrombus ^b^			
Present	15 (62.5%)	13 (72.3%)	*p* = 0.742
Absent	9 (37.5%)	5 (27.7%)	
Thrombus ≥ 1 mm ^b^			
Present	4 (16.6%)	5 (27.7%)	*p =* 0.462
Absent	20 (83.4%)	13 (72.3%)	

MN = mononuclear leucocytes. PMN = polymorphonuclear leucocytes. DAD = diffuse alveolar damage. NLR = neutrophil–lymphocyte ratio. *n* = number of patients. Categorical variable expressed by absolute number (frequency). Continuous variables expressed by mean ± standard deviation. ^a^ Chi-square. ^b^ Fisher’s exact test. *p*-valor  <  0.05 considered statistically significant. Reference values: thrombocytopenia < 150,000 per mm^3^ of blood, leukocytosis > 10,000 per mm^3^ of blood and neutrophilia > 8000 per mm^3^ of blood. The results were performed 24 h before death.

**Table 7:** Elevated D-dimer levels on Day 1 in patients from the first and second COVID-19 waves.

D-Dimer Values of Day 1 (*n* = 30)	First Wave (*n* = 17)	Second Wave (*n* = 13)
Value > 500 (ng/mL)	16/17 (94.1%)	10/13 (76.9%)

Categorical variable expressed as absolute number (frequency). ng/mL = nanograms per milliliter of blood.

**Table 8:** Clinical and microscopic findings of cases with evidence of NETS.

Clinical Information	Value
Number of cases with evidence of NETs	10
Age range (years)	58.4 ± 11.5
Hospitalization range (days)	18.3 ± 8.4
Patients from first wave of COVID-19	3 cases (30%)
Patients from second wave of COVID-19	7 cases (70%)
**Microscopic Findings**	**Value**
Presence of microthrombi	8 (80%)
Moderate- intense MN alveolar infiltrate	10 (100%)
Moderate- intense PMN aveolar infiltrate	8 (80%)

MN = mononuclear leucocytes. PMN = polymorphonuclear leucocytes. Categorical variables expressed as absolute number (frequency).

**Table 9:** Hematology results of cases with evidence of NETS.

Variables	Day 1 (*n* = 10)	Day F (*n* = 9)
Thrombocytopenia	5 (50%)	2 (22.2%)
Leukocytosis	3 (30%)	9 (100%)
Neutrophilia	4 (40%)	9 (100%)
Mean Neutrophil Count	8876/μL	20,294/μL
Mean Leukocyte Count	10,470/μL	23,200/μL
D-dimer > 500 ng/mL	–	5/7 (71.4%)
NLR	15.1 ± 12.2	26.6 ± 25.2 (*n* = 8)

Categorical variable expressed as absolute number (frequency). Continuous variables expressed by mean ± standard deviation. /μL = per microliter of blood. ng/mL = nanograms per milliliter of blood. Reference values: plaquetopenia < 150,000 per mm^3^ of blood, leukocytosis > 10,000 per mm^3^ of blood, leucopenia < 4000 per mm^3^ of blood, and neutrophilia > 8000 per mm^3^ of blood. NLR = neutrophil-lymphocyte ratio.

## Data Availability

The data for all patients and samples included in this study are described in Supplemental Tables S1 and S2.

## Supplementary Materials

The following supporting information can be downloaded at: https://www.mdpi.com/article/10.3390/ijms26168059/s1.

## Abbreviations

The following abbreviations are used in this manuscript:

SARS-CoV-2	Severe Acute Respiratory Syndrome Coronavirus 2
COVID-19	Coronavirus disease 2019
CRP	C-reactive protein
NETs	Neutrophil extracellular traps
MPO	Myeloperoxidase
ICU	Intensive care unit
DAD	Diffuse alveolar damage
sd	Standard deviation
Day 1	First day of hospitalization
Day F	Final 24 h of hospitalization or prior to death
PMN	Polymorphonuclear leucocytes
NLR	Neutrophil-to-lymphocyte ratio
MN	Mononuclear leucocytes
Group 1	Patients from the first wave
Group 2	Patients from the second wave
cit-H3	Citrullinated histone H3
H&E	Hematoxylin–Eosin
FFPE	Formalin-fixed and paraffin-embedded
CONEP	National Research Ethics Committee
RT-qPCR	Reverse Transcription Quantitative Polymerase Chain Reaction
TMA	Tissue Microarray
VOC	Variants of concern
