# Supplementary material for: Neutrophil Extracellular Trap Markers in Post Mortem Lung Biopsies from COVID-19 Patients
Source: Int J Mol Sci. 2025 Aug 20;26(16):8059. doi: 10.3390/ijms26168059 (PMC12386323; doi:10.3390/ijms26168059)
Supplement: Supplementary file 1 [file ijms-26-08059-s001.zip › ijms-3739542-supplementary.pdf]

Supplementary Table S1 – Clinical and histopathological data.

	**Clinical characteristics**					**Histopatologic analysis**									
						Alveolar compartment				Intestitial compartment			Vascular compartment		
	**Gender**	**Age (years)**	**Lenth of hospitalization before death (days)**	**Period of death**	**DAD - phase**	**Alveolar MN infiltrate**	**Alveolar PNM infiltrate**	**Hyaline membrane**	**Edema**	**Interstiticial MN infiltrate**	**Interstitical PNM infiltrate**	**Fibrosis**	**Microthromi**	**Additional findings**	**NETS (citH3+ and MPO+)**
**Case 1**	Female	87	6	First Wave	Exudative phase	Mild	Absent	Present	Mild	Mild	Mild	Mild	Absent		
**Case 2**	Male	53	12	First Wave	Proliferative phase	Mild	Mild	Present	Absent	Intense	Mild	Moderate	Present	Thrombus with neutrophils, capillaritis, thrombus >0.9 mm.	
**Case 3**	Female	85	23	First Wave	Exudative phase	Moderate	Mild	Present	Moderate	Moderate	Moderate	Mild	Present	Capilaritis.	
**Case 4**	Male	73	38	First Wave	Proliferative phase	Intense	Moderate	Present	Mild	Moderate	Mild	Moderate	Present	Thrombus with neutrophils, capillaritis.	
**Case 5**	Male	80	23	First Wave	Exudative phase	Mild	Absent	Present	Mild	Mild	Mild	Absent	Present	Thrombus >0.9 mm.	
**Case 6**	Male	81	8	First Wave	Exudative phase	Mild	Mild	Absent	Mild	Mild	Absent	Absent	Absent		

**Case 7**	Female	70	20	First Wave	Fibrotic phase	Moderate	Mild	Present	Mild	Intense	Moderate	Intense	Present	Thrombus with neutrophils, capillaritis, thrombus >0.9 mm.	
**Case 8**	Male	86	6	First Wave	Proliferative phase	Moderate	Mild	Present	Absent	Intense	Absent	Intense	Present	Capilaritis.	
**Case 9**	Male	46	8	First Wave	Proliferative phase	Mild	Absent	Present	Mild	Moderate	Mild	Moderate	Present	Thrombus with neutrophils, capillaritis.	
**Case 10**	Female	93	6	First Wave	Proliferative phase	Moderate	Mild	Present	Mild	Moderate	Mild	Intense	Absent		
**Case 11**	Male	64	1	First Wave	Exudative phase	Mild	Absent	Present	Moderate	Mild	Absent	Absent	Absent		
**Case 12**	Female	75	8	First Wave	Proliferative phase	Intense	Intense	Present	Mild	Mild	Mild	Mild	Present	Thrombus with neutrophils, capillaritis.	Evidence of NETS
**Case 13**	Male	64	9	First Wave	Exudative phase	Mild	Absent	Present	Mild	Mild	Mild	Absent	Absent		
**Case 14**	Male	57	11	First Wave	Exudative phase	Intense	Mild	Present	Intense	Moderate	Mild	Moderate	Present	Thrombus with neutrophils, capillaritis,	

														thrombus >0.9 mm.	
**Case 15**	Female	81	14	First Wave	Exudative phase	Mild	Absent	Absent	Mild	Mild	Absent	Absent	Absent		
**Case 16**	Male	57	39	First Wave	Fibrotic phase	Moderate	Mild	Present	Mild	Intense	Mild	Moderate	Present	Capilaritis.	Evidence of NETS
**Case 17**	Female	60	24	First Wave	Proliferative phase	Intense	Mild	Present	Moderate	Intense	Mild	Intense	Present		
**Case 18**	Male	75	9	First Wave	Proliferative phase	Intense	Mild	Present	Mild	Moderate	Mild	Mild	Present		
**Case 19**	Male	70	16	First Wave	Fibrotic phase	Moderate	Mild	Absent	Moderate	Moderate	Mild	Intense	Absent		
**Case 20**	Male	57	24	First Wave	Fibrotic phase	Moderate	Mild	Present	Absent	Moderate	Mild	Intense	Absent		Evidence of NETS
**Case 21**	Male	86	8	First Wave	Exudative phase	Mild	Absent	Absent	Mild	Absent	Absent	Absent	Present		
**Case 22**	Male	83	16	First Wave	Proliferative phase	Moderate	Absent	Absent	Mild	Moderate	Mild	Intense	Absent		
**Case 23**	Female	72	30	First Wave	Proliferative phase	Mild	Intense	Present	Mild	Moderate	Moderate	Moderate	Present	Capilaritis.	
**Case 24**	Female	72	22	First Wave	Proliferative phase	Moderate	Mild	Present	Moderate	Moderate	Mild	Moderate	Present		
**Case 25**	Male	57	14	Second wave	Fibrotic phase	Mild	Mild	Present	Mild	Moderate	Moderate	Intense	Absent	Capilaritis.	

**Case 26**	Male	54	10	Second wave	Proliferative phase	Intense	Intense	Present	Moderate	Moderate	Intense	Moderate	Absent	Capilaritis.	Evidence of NETS
**Case 27**	Male	79	18	Second wave	Fibrotic phase	Moderate	Intense	Present	Mild	Moderate	Moderate	Intense	Present	Thrombus with neutrophils, capillaritis.	
**Case 28**	Female	51	22	Second wave	Fibrotic phase	Moderate	Moderate	Present	Mild	Moderate	Mild	Moderate	Present	Thrombus with neutrophils, capillaritis, thrombus >0.9 mm.	Evidence of NETS
**Case 29**	Male	51	23	Second wave	Fibrotic phase	Moderate	Intense	Present	Mild	Moderate	Moderate	Moderate	Present	Thrombus with neutrophils, capillaritis.	Evidence of NETS
**Case 30**	Male	77	24	Second wave	Fibrotic phase	Intense	Intense	Present	Moderate	Moderate	Moderate	Intense	Present	Capilaritis.	
**Case 31**	Male	50	33	Second wave	Fibrotic phase	Mild	Absent	Present	Mild	Moderate	Absent	Moderate	Absent		
**Case 32**	Male	64	8	Second wave	Proliferative phase	Moderate	Intense	Present	Moderate	Mild	Moderate	Moderate	Present	Thrombi with neutrophils, capillaritis, thrombus >0.9 mm.	Evidence of NETS

**Case 33**	Male	58	25	Second wave	Fibrotic phase	Moderate	Mild	Present	Mild	Moderate	Moderate	Intense	Present	Thrombi with neutrophils, thrombus >0.9 mm.	
**Case 34**	Female	59	14	Second wave	Exudative phase	Intense	Intense	Present	Moderate	Mild	Moderate	Absent	Present	Capilaritis.	Evidence of NETS
**Case 35**	Male	45	15	Second wave	Exudative phase	Moderate	Mild	Absent	Absent	Mild	Mild	Mild	Present	Thrombus with neutrophils, capillaritis, thrombus >0.9 mm.	
**Case 36**	Male	53	10	Second wave	Exudative phase	Moderate	Intense	Present	Intense	Mild	Intense	Absent	Present	Thrombus with neutrophils, capillaritis, thrombus >0.9 mm.	Evidence of NETS
**Case 37**	Female	63	25	Second wave	Proliferative phase	Moderate	Intense	Present	Moderate	Mild	Moderate	Mild	Present	Capilaritis.	Evidence of NETS
**Case 38**	Male	40	21	Second wave	Exudative phase	Mild	Absent	Present	Mild	Moderate	Moderate	Absent	Present	Thrombus with neutrophils.	
**Case 39**	Male	30	12	Second wave	Exudative phase	Moderate	Moderate	Absent	Intense	Moderate	Moderate	Mild	Present	Capilaritis.	

**Case 40**	Male	52	14	Second wave	Fibrotic phase	Moderate	Moderate	Absent	Mild	Moderate	Mild	Intense	Present
**Case 41**	Male	83	17	Second wave	Fibrotic phase	Moderate	Moderate	Absent	Mild	Mild	Mild	Intense	Absent
**Case 42**	Male	65	13	Second wave	absent	Absent	Absent	Absent	Absent	Absent	Absent	Mild	Absent

PNM= polymorphonuclear leukocyte, MNL= mononuclear leukocyte,

Supplementary Table S2 – Laboratory data.

	Blood exams							
	24 hours before death				D1 of hospitalization			
	**Leucocytes**	**Neutrophils**	**Platelets**	**D-dimer**	**Leucocytes**	**Neutrophils**	**Platelets**	**D-dimer**
**Case 1**	15000	13500	239000		16200	15714	379000	
**Case 2**	50200	45180	318000	7384	11000	9790	311000	425
**Case 3**	9900	8118	481000		9900	7623	282000	
**Case 4**	22000	20900	356000		17600	15488	180000	
**Case 5**	9400	7802	142000	4057	4700	3807	112000	816
**Case 6**	31400	30458	172000		14300	13299	276000	
**Case 7**	15880	11910		1129	7380	5850	218000	4160
**Case 8**	8900	7921	99000		8300	7470	131000	
**Case 9**	19500	17550	175000		3800	2926	131000	594
**Case 10**	15600	14820	76000		7100	6319	111000	6544
**Case 11**	10600	9222	439000		9300	7998	354000	61237
**Case 12**	34900	32108	155000		15300	13311	176000	1652
**Case 13**	9800	7644	232000		4000	2760	162000	1599
**Case 14**	15300	12699	15000	6561	14500	13775	186000	628
**Case 15**	28900	27455	141000		17000	13430	196000	
**Case 16**	15800	13904	395000		7400	5476	146000	
**Case 17**	8400	8148	58000		8100	7452	244000	3701
**Case 18**	19100	16426	336000		13100	11397	200000	152134
**Case 19**	15500	14725	102000		11600	10904	149000	1848
**Case 20**	29100	25317	287000		5900	4897	143000	745
**Case 21**	4700	4136	132000		4200	3486	127000	848
**Case 22**	30700	28858	419000	1564	9500	8930	166000	1010
**Case 23**	32200	30590	190000	5058	9000	7920	225000	575
**Case 24**	7900	7110	180000	7550	5900	4720	219000	1975

**Case 25**	13500	11880	366000		4500	4005	178000	290
**Case 26**	22000	20680	363000		8500	6290	117000	290
**Case 27**	34500	32775	287000		4500	3420	110000	1822
**Case 28**			528000		7300	6351	250000	
**Case 29**	14400	10800	377000		18200	16562	305000	342
**Case 30**	11300	8927	274000		10000	8800	227000	774
**Case 31**	34900	30363	143000		11100	10101	238000	6085
**Case 32**	16400	15252	599000		6900	6141	300000	934
**Case 33**	7300	5037	424000		9800	8918	217000	
**Case 34**	34300	33614	92000		8900	6764	162000	
**Case 35**	38300	32555	78000		19000	17480	26400	
**Case 36**	25500	23205	336000		15000	12300	193000	563
**Case 37**	16400	15744	105000		11300	10622	17300	887
**Case 38**	23900	23422	182000	984	4300	3483	147000	896
**Case 39**	15400	12320	580000		7900	6399	294000	1839
**Case 40**	28900	24276	371000		28900	26299	113000	5536
**Case 41**	4900	4557	690000		2900	2146	530000	
**Case 42**	21000	16590	239000		8300	7802	166000	6190

Leukocyte, neutrophil, and platelet counts are expressed as the total number of cells per microliter (cells/μL) of peripheral blood. D-dimer levels are reported in nanograms per milliliter (ng/mL) of blood.
